# Hipertensão em Populações Indígenas: Desafios Diagnósticos e Nuances da Transição Epidemiológica

**DOI:** 10.36660/abc.20260138

**Published:** 2026-06-16

**Authors:** Letícia Lemos Belo, Maria Clara Gomes Pontes, Thalisson Gonçalves Almeida, Tales Gabriel Ferro de Oliveira, Pedro Pereira Tenório

**Affiliations:** 1 Universidade Federal do Vale do São Francisco Paulo Afonso BA Brasil Universidade Federal do Vale do São Francisco (UNIVASF), Paulo Afonso, BA - Brasil

**Keywords:** Hipertensão, Povos Indígenas, Fatores de Risco, Perfil de Saúde, Saúde de Populações Indígenas

**Prezado editor,**

Submetemos esta carta para tecer considerações sobre o relevante artigo intitulado: *"Prevalência de Hipertensão Arterial Sistêmica e Fatores Associados em Indígenas Atendidos em um Ambulatório Especializado no Sul do Brasil".*^1^ O estudo aborda uma lacuna crítica no conhecimento epidemiológico das populações originárias da região Sul, oferecendo uma visão valiosa sobre a transição epidemiológica que as atinge. O trabalho destaca-se por sua importância social e técnica ao preencher um vazio de dados sobre indígenas no Sul do Brasil, grupo que enfrenta desafios únicos de acesso à saúde e preservação cultural.

A utilização de dados do segundo maior ambulatório de média e alta complexidade para indígenas do país confere robustez à análise de pacientes em acompanhamento especializado. A forte associação encontrada entre a hipertensão arterial sistêmica (HAS) e a diabetes mellitus (IC95 1,48-3,66) reforça a necessidade de estratégias de manejo integrado para essas doenças crônicas. O destaque para o envelhecimento, e situação conjugal como fatores associados permite uma compreensão mais holística dos riscos nessa população. No entanto, o sexo, as condições de moradia, a escolaridade, tabagismo, presença de dislipidemia, tabagismo, consumo de bebida alcoólica e prática de atividade física, não terem alcançado significância estatística (p<0,05) são tendências que merecem mais estudos, pois são abordados na Diretriz Brasileira de Hipertensão Arterial de 2025.

Apesar dos méritos do estudo, alguns pontos merecem reflexão para futuras investigações e para a interpretação cautelosa dos dados. Como os autores reconhecem, a coleta baseada em dados secundários de prontuários pode levar ao subdiagnóstico ou a heterogeneidade de critérios. Além disso, a amostra por conveniência em um ambulatório especializado pode não refletir a realidade das aldeias mais isoladas. A falta de dados sobre como são realizados os diagnósticos também configura uma limitação, já que ferramentas padronizadas como monitoramento residencial da pressão arterial ou monitoramento ambulatorial da pressão arterial são imprescindíveis para monitorização e classificação da HAS, tendo em vista a possibilidade de interferências no diagnóstico, como o "efeito do jaleco branco", que podem superestimar a real prevalência de HAS na população indígena quando não utilizado, posteriormente, um métodos fora de consultório para confirmação diagnóstica.^2^

O aprofundamento sobre como a urbanização e a perda de hábitos tradicionais impactam especificamente esses comportamentos poderia enriquecer a discussão sobre prevenção, já que a Diretriz Brasileira de Hipertensão Arterial de 2025 descreve uma relação de prevalência mais alta de HAS em indígenas que residem em áreas urbanas em comparação com indígenas que vivem em áreas de menor urbanização.^3^ Ademais, observa-se uma baixa prevalência de atividade física (7,5%) aliado a uma parcela significativa de tabagistas e ex-tabagistas (26,6%) entre a população indígena segundo os dados obtidos no artigo, o que pode atentar para um processo de transição epidemiológica entre esses povos, principalmente no que tange hábitos alimentares, em que, como abordado no artigo, a proximidade com os centros urbanos acarreta a uma maior facilidade e exposição aos alimentos ultraprocessados que são ricos em sódio. Logo, seria importante abordar nos futuros trabalhos a relação entre o consumo exacerbado de industrializados e alimentos hipercalóricos por parte dos povos indígenas, visando quantificar a maior prevalência de HAS e doenças cardiovasculares nessas comunidades.^4^

Por fim, o desenho transversal impede o estabelecimento de causalidade direta (causalidade reversa), sendo necessários estudos longitudinais para monitorar a progressão da HAS e a eficácia das intervenções da atenção primária à saúde (APS). Todavia, o estudo cumpre o papel essencial de ratificar a HAS como um problema de saúde pública urgente entre indígenas. Os achados sublinham a importância de uma APS fortalecida e culturalmente sensível, capaz de atuar no diagnóstico precoce e na mitigação dos fatores de risco associados à modernização e ao envelhecimento.
