# Supplementary material for: Hipertensão em Populações Indígenas: Desafios Diagnósticos e Nuances da Transição Epidemiológica
Source: Arq Bras Cardiol. 2026 Jun 16;123(5):e20260138. [Article in Portuguese] doi: 10.36660/abc.20260138 (PMC13399110; doi:10.36660/abc.20260138)
Supplement: Translation

# Translation: Hypertension in Indigenous Populations: Diagnostic Challenges and Nuances of the Epidemiological Transition

**DOI:** 10.36660/abc.20260138i

**Authors:** Letícia Lemos Belo, Maria Clara Gomes Pontes, Thalisson Gonçalves Almeida, Tales Gabriel Ferro de Oliveira, Pedro Pereira Tenório

**Affiliations:** 1 Paulo Afonso BA Brazil Universidade Federal do Vale do São Francisco (UNIVASF), Paulo Afonso, BA - Brazil

**Keywords:** Hypertension, Indigenous Peoples, Risk Factors, Health Profile, Health of Indigenous Peoples

**Dear editor,**

We are submitting this letter to offer our considerations on the relevant article entitled: "Prevalence of Systemic Arterial Hypertension and Associated Factors in Indigenous People Attended at a Specialized Outpatient Clinic in Southern Brazil".^1^ The study addresses a critical gap in the epidemiological knowledge of the native populations of the Southern region, offering valuable insight into the epidemiological transition affecting them. The work stands out for its social and technical importance in filling a data void on indigenous people in Southern Brazil, a group that faces unique challenges in accessing healthcare and preserving their culture.

The use of data from the second-largest medium and high complexity outpatient clinic for indigenous people in the country lends robustness to the analysis of patients under specialized care. The strong association found between systemic arterial hypertension (SAH) and diabetes mellitus (95% CI 1.48-3.66) reinforces the need for integrated management strategies for these chronic diseases. The emphasis on aging and marital status as associated factors allows for a more holistic understanding of the risks in this population. However, the fact that sex, housing conditions, education level, smoking, presence of dyslipidemia, alcohol consumption, and physical activity did not reach statistical significance (p<0.05) is a trend that deserves further study, as they are addressed in the 2025 Brazilian Hypertension Guidelines.

Despite the merits of the study, some points deserve reflection for future investigations and for the cautious interpretation of the data. As the authors acknowledge, data collection based on secondary medical records can lead to underdiagnosis or heterogeneity of criteria. Furthermore, convenience sampling in a specialized outpatient clinic may not reflect the reality of more isolated villages. The lack of data on how diagnoses are made also constitutes a limitation, since standardized tools such as home blood pressure monitoring or ambulatory blood pressure monitoring are essential for monitoring and classifying hypertension, considering the possibility of diagnostic interferences, such as the "white coat effect," which can overestimate the true prevalence of hypertension in the indigenous population when out-of-office methods are not subsequently used for diagnostic confirmation.^2^

A deeper understanding of how urbanization and the loss of traditional habits specifically impact these behaviors could enrich the discussion on prevention, since the 2025 Brazilian Hypertension Guidelines describe a higher prevalence of hypertension in indigenous people residing in urban areas compared to indigenous people living in less urbanized áreas.^3^ Furthermore, a low prevalence of physical activity (7.5%) is observed, along with a significant proportion of smokers and former smokers (26.6%) among the indigenous population, according to the data obtained in the article. This may point to an epidemiological transition process among these peoples, especially regarding dietary habits, where, as discussed in the article, proximity to urban centers leads to greater ease and exposure to ultra-processed foods that are high in sodium. Therefore, it would be important to address in future studies the relationship between the excessive consumption of processed and high-calorie foods by indigenous peoples, aiming to quantify the higher prevalence of hypertension and cardiovascular diseases in these communities.^4^

Finally, the cross-sectional design prevents the establishment of direct causality (reverse causality), making longitudinal studies necessary to monitor the progression of hypertension and the effectiveness of Primary Health Care (PHC) interventions. However, the study fulfills the essential role of ratifying hypertension as an urgent public health problem among indigenous people. The findings underscore the importance of a strengthened and culturally sensitive PHC system, capable of acting in early diagnosis and mitigating risk factors associated with modernization and aging.
